# Contrasting patterns of sequence variation in steelhead populations reflect distinct evolutionary processes

**DOI:** 10.1111/eva.13623

**Published:** 2023-12-11

**Authors:** Stuart Willis, D. Katharine Coykendall, Matthew R. Campbell, Shawn Narum

**Affiliations:** ^1^ Hagerman Genetics Lab Columbia River Inter‐Tribal Fish Commission Hagerman Idaho USA; ^2^ Eagle Fish Genetics Lab Pacific States Marine Fisheries Commission Eagle Idaho USA; ^3^ Eagle Fish Genetics Lab Idaho Department of Fish and Game Eagle Idaho USA

**Keywords:** fisheries management, genomics/proteomics, population genetics – empirical

## Abstract

Multiple evolutionary processes influence genome‐wide allele frequencies and quantifying effects of genetic drift, and multiple forms of selection remain challenging in natural populations. Here, we investigate variation at major effect loci in contrast to patterns of neutral drift across a wide collection of steelhead (*Oncorhynchus mykiss*) populations that have declined in abundance due to anthropogenic impacts. Whole‐genome resequencing of 74 populations of steelhead revealed genome‐wide patterns (~8 million SNPs) consistent with expected neutral population structure. However, allelic variation at major effect loci associated with adult migration timing (chromosome 28: *GREB1L*/*ROCK1*) and age at maturity (chromosome 25: *SIX6*) reflected how selection has acted on phenotypic variation in contrast with neutral structure. Variation at major effect loci was influenced by evolutionary processes with differing signals between the strongly divergent Coastal and Inland lineages, while allele frequencies within and among populations within the Inland lineage have been driven by local natural selection as well as recent anthropogenic influences. Recent anthropogenic effects appeared to have influenced the frequency of major effect alleles including artificial selection for specific traits in hatchery stocks with subsequent gene flow into natural populations. Selection from environmental factors at various scales has also likely influenced variation for major effect alleles. These results reveal evolutionary mechanisms that influence allele frequencies at major effect loci that are critical for conservation of phenotypic traits and life history variation of this protected species.

## 1 INTRODUCTION

Diversity in life history traits and trophic adaptations are believed to increase the resilience of populations to periodic and stochastic environmental variation (Hoelzel et al., 2019). This preserves the ecological roles exhibited by these populations in a phenomenon called ‘portfolio effects’, where alleles and species are assets that, when diversified, lead to a more resilient and stable ecosystem (Figge, 2004; Moore et al., 2014; Schindler et al., 2010). Therefore, a major goal of conservation management is to identify and track this diversity, as well as understand recent and prehistorical evolutionary context, including genetic architecture and interaction with phenotypic plasticity (Funk et al., 2012; Hoelzel et al., 2019; Skovmand et al., 2018). This goal has been expedited by ongoing improvements in sequencing technologies, which have allowed researchers to survey genomes in greater numbers and extent so as to identify gene regions associated with quantifiable phenotypic variants with higher power and precision (Hoban et al., 2016). However, while identifying genetic variants associated with important traits may now be routinely accessible, fully resolving causal mutations and pleiotropic interactions, understanding their complex evolutionary history, and developing strategies and protocols to utilize these in conservation management, remain challenging (Kardos & Shafer, 2018; Pearse, 2016; Waples & Lindley, 2018).

Rainbow trout (*Oncorhynchus mykiss*), the anadromous forms of which are called steelhead, exhibit an impressive array of life history diversity. This includes notable and important variation in adult migration phenology (run timing), age at maturity (age at first return migration), propensity for residency or anadromy, precocious sexual maturation, and iteroparity (repeat spawning) (Busby et al., 2000; Carlson & Seamons, 2008; Quinn et al., 2011). Many of these traits are highly variable both among and within phylogeographic lineages and local populations (Busby et al., 2000), and are part of the portfolio of life history diversity that assist populations in persisting through natural and anthropogenic environmental change (Quinn et al., 2016). For several traits known to reflect heritable variation in *O. mykiss*, sequencing of individuals with known phenotypic states has identified genomic regions significantly associated with this variation, including a region on chromosome 28 (chr. 28) containing two genes, *GREB1L* and *ROCK1*, strongly associated with migration phenology (Hess, Ackerman, et al., 2016; Micheletti, Hess, et al., 2018; Prince et al., 2017), and a region on chromosome 25 (chr. 25) including the gene *SIX6* that is associated with age at maturity (Waters et al., 2021). While progress has been made to identify the strength and pattern of association of specific genetic markers in these regions (Ford et al., 2020; Thompson et al., 2019; Thompson et al., 2020; Waples et al., 2022; Willis et al., 2020), much remains to be resolved about the effects of these loci on adult migration timing and age at maturity, in particular, the demographic and evolutionary context and history of these genomic regions.

For the diverse populations of steelhead in the Columbia River Basin, maintaining genetic and phenotypic variation is necessary for long‐term persistence of this species. Steelhead in the Columbia River make freshwater migrations up to 1100 km, with many populations listed under the U.S. Endangered Species Act (ESA) and actively managed to ensure persistence (Busby et al., 2000). Migration itself is a challenging proposition for these steelhead, as they contend with thermal extremes and numerous physical barriers, including up to nine hydroelectric dams, to reach spawning tributaries (Keefer et al., 2008; Keefer, Jepson, et al., 2018). Columbia River steelhead derive from two phylogeographic lineages: a Coastal lineage (*O. mykiss irideus*), found primarily west of the Cascade Range and elsewhere on the Pacific Coast, and an Inland Lineage (*O. mykiss gairdneri*), exclusive to the Columbia River Basin east of the Cascades, with at least one intermediate population in the Klickitat River (Blankenship et al., 2011; Micheletti, Matala, et al., 2018). In addition, the Columbia River Basin has an extensive history of hatchery stocking, of both anadromous and resident forms, sometimes with strains that are highly distinct genetically and/or phenotypically to the local stock. Several of these anadromous hatchery strains have well‐known and unique phenotypic characteristics (e.g., Bowersox et al., 2019), due to both deliberate and inadvertent selections for particular traits. Perhaps one of the most notable of these is the Skamania Hatchery stock, which has been outplanted in numerous locales throughout the basin, and is known for early return times for adults (late spring, early summer) and older ages at return (2‐year ocean duration) (Ayerst, 1976; Chilcote et al., 1986; Crawford, 1979; Hess, Ackerman, et al., 2016).

Within this complex demographic and evolutionary context, adult migration timing and age at maturity in Columbia River steelhead have evolved as a result of both natural and anthropogenic forces. For example, Coastal Lineage steelhead exhibit adult migration into freshwater both early (summer) and late (winter), often spawning in proximity within the same tributaries the following spring, while stocks in the Inland Lineage migrate exclusively early (in summer through fall) before spawning in spring. Notably, however, Inland lineage steelhead have a high frequency of alleles and haplotypes that are strongly associated with late migration timing in the Coastal Lineage (Collins et al., 2020; Willis et al., 2020), indicating that the genetic architecture of early versus late adult migration timing may differ for the two major lineages. Variation in adult migration timing of the Inland Lineage within this constrained summer–fall period is nonetheless broadly associated with the same region on chr. 28, suggesting that other variants within this region and/or other regions may additionally influence migration phenology in these fish (Willis et al., 2020). As Inland lineage steelhead generally have more challenges to migration than their Coastal lineage counterparts, summer–fall migration into freshwater has been hypothesized to be an adaptation to utilize interior spawning tributaries that differ from coastal rivers in the timing of greatest accessibility and suitability (Quinn et al., 2016). However, summer–fall temperatures and flows also are among those predicted to change the most significantly due to anthropogenic climate change (Keefer, Jepson, et al., 2018; Quinn et al., 2016), making Inland lineage stocks, most of which are currently listed as threatened or endangered under the ESA, especially susceptible to extinction. In contrast to migration timing, there is generally more variation in age at maturity both within stocks and regionally (Busby et al., 2000), though it is notable that older (longer ocean duration) fish tend to be larger and more susceptible to in‐river fisheries, and indeed the catch of (2 + −ocean) steelhead is used to regulate these fisheries (Copeland et al., 2017). Since age at maturity is significantly associated with the chr. 25 gene *SIX6* in a sex‐specific manner, this selective pressure has the potential to alter both age at maturity variation and sex ratios in the Inland lineage stocks that migrate during this period (Willis et al., 2020).

The intersection of contemporary selective forces and evolutionary history makes understanding the association and interactions of genomic background and trait‐associated genomic regions an important area of research for steelhead (Waples et al., 2022). Unlike previous studies that used curated single‐nucleotide polymorphisms (SNPs) or reduced representation genomic libraries, here we examine variation from ‘normalized pool‐seq’ with individually barcoded samples, a form of low‐coverage whole‐genome sequencing (mean coverage per individual <1×; hereafter lcWGS), from representatives of numerous stocks distributed throughout the Columbia River Basin that have been influenced by both natural and anthropogenic forces. Using these extensive data, we addressed the following questions: (1) Does neutral variation from lcWGS support previous estimates of genetic structure of steelhead? (2) Does variation at major effect loci for adult migration timing (*GREB1L*/*ROCK1*) and age at maturity (*SIX6*) contrast from neutral variation? (3) Have anthropogenic actions produced patterns inconsistent with the natural variation in these large effect loci?

## 2 METHODS

### 2.1 Steelhead collections and DNA library preparation

**FIGURE 1:**
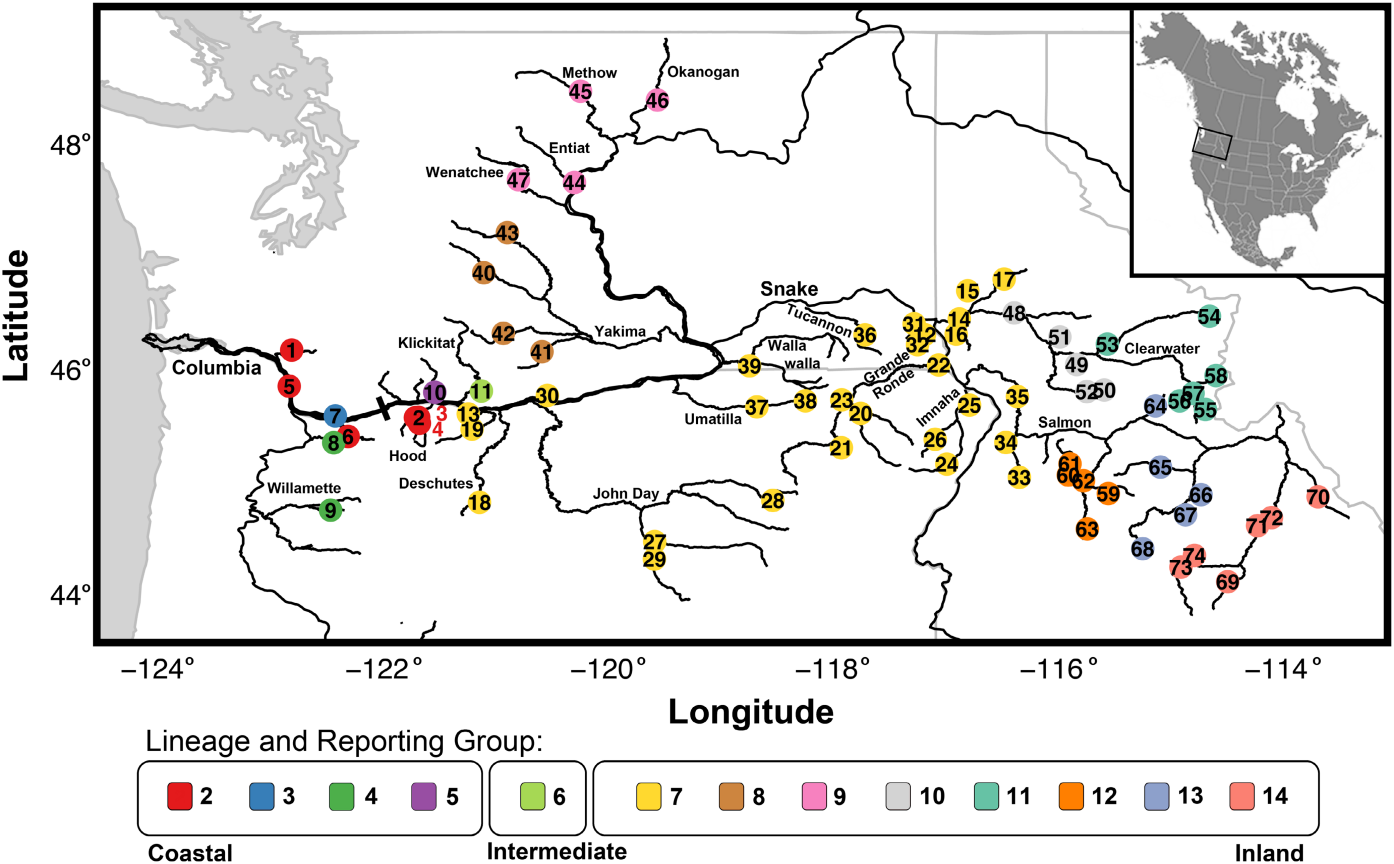
Collections of 74 steelhead populations that were included for whole‐genome resequencing. Point labels refer to collections and correspond to Table 1. Colors reflect reporting groups utilized for fisheries management, which are themselves based on genetic affinity. Reporting groups each belong to a steelhead lineage, which are labeled and grouped by lineage beneath the plot (reporting groups 2 through 14; reporting group 1, ‘Outside Columbia’ is not included). Bonneville, the first major barrier to inland migration, is indicated with a solid bar.

Seventy‐four collections of steelhead from geographic locations throughout the Columbia River were sequenced to represent genetically distinct populations of steelhead (Figure 1). Collections contained 30–119 individuals (mean 65.9) with a total of 4873 individual samples across collections. Where possible, individuals were chosen to represent several proximal collection localities and years to encapsulate representative genetic diversity (Table 1; Table S1). Collections were putatively from natural origin, anadromous populations, with the exception of five anadromous hatchery stocks from Skamania Fish Hatchery (Washington Department of Fish and Wildlife; Washougal, WA), Parkdale Fish Hatchery (Confederated Tribes of Warm Springs/Oregon Department of Fish and Wildlife; Mt Hood, OR), Dworshak Fish Hatchery (Nez Perce Tribe and U.S. Fish & Wildlife; Orofino, ID), Pahsimeroi Fish Hatchery (Idaho Department of Fish and Game; May, ID), and Sawtooth Fish Hatchery (Idaho DFG; Stanley, ID).

**TABLE 1:** Collections of 74 steelhead populations from the Columbia Basin that were included for whole‐genome resequencing.

No.	Lineage	Subbasin	Localities or stocks	Run	N	%Cov@ 10reads^a^
1	Coastal	Cowlitz	Cowlitz, Coweeman	winter	48	67%
2	Coastal	Hood	East Fork Hood	winter	46	67%
3	Coastal	Hood	Parkdale Fish Hatchery	winter	83	67%
4	Coastal	Hood	West Fork Hood	summer	79	68%
5	Coastal	Lewis	East Fork Lewis	unk.	78	77%
6	Coastal	Sandy	Sandy Hatchery, Bull Run	unk.	87	73%
7	Coastal	Washougal River	Skamania Hatchery	summer	60	68%
8	Coastal	Willamette	Eagle	winter	61	66%
9	Coastal	Willamette	Little Rock	unk.	50	65%
10	Coastal	White Salmon	Big White Salmon R	unk.	95	69%
11	Interm.	Klickitat	*Several*	unk.	75	70%
12	Inland	Asotin	Asotin	summer	60	75%
13	Inland	Columbia Gorge	Mill Creek	unk.	96	67%
14	Inland	Clearwater	Lapwai Cr, Mission Cr	summer	119	66%
15	Inland	Clearwater	Little Bear Creek	unk.	46	66%
16	Inland	Clearwater	Sweetwater Creek	summer	51	61%
17	Inland	Clearwater	WF Potlatch River	summer	50	66%
18	Inland	Deschutes	*Several*	summer	84	71%
19	Inland	Fifteenmile Creek	Fifteenmile Cr	unk.	92	67%
20	Inland	Grand Ronde	Big Canyon	summer	89	68%
21	Inland	Grand Ronde	Catherine	unk.	90	68%
22	Inland	Grand Ronde	Joseph	summer	88	70%
23	Inland	Grand Ronde	Upper Grande Ronde	unk.	58	68%
24	Inland	Imnaha	Gumboot Creek	summer	38	61%
25	Inland	Imnaha	Lightning Cr	summer	76	69%
26	Inland	Imnaha	LittleSheep	unk.	75	71%
27	Inland	John Day	JDR Upper mainstem	unk.	70	68%
28	Inland	John Day	*Several*	summer	53	73%
29	Inland	John Day	Murderers	unk.	96	66%
30	Inland	Columbia Gorge	Rock	unk.	91	67%
31	Inland	Tucannon	Alpowa Creek	summer	53	67%
32	Inland	Asotin	George Creek	summer	58	67%
33	Inland	Salmon	Little Salmon R, etc.	summer	91	65%
34	Inland	Salmon	Rapid River	unk.	78	73%
35	Inland	Salmon	Whitebird	summer	50	70%
36	Inland	Tucannon	Tucannon	summer	42	67%
37	Inland	Umatilla	Minthorn	summer	74	71%
38	Inland	Umatilla	Umatilla	summer	70	73%
39	Inland	Walla Walla	*Several*	unk.	30	70%
40	Inland	Yakima	Naches ‐Nile Creek	summer	38	64%
41	Inland	Yakima	Satus	unk.	67	68%
42	Inland	Yakima	Toppenish	unk.	91	67%
43	Inland	Yakima	Teanaway, Big Cr, Roza	unk.	71	69%
44	Inland	Entiat	Entiat	unk.	33	64%
45	Inland	Methow	Winthrop	summer	93	65%
46	Inland	Okanogan	*Several*	summer	59	59%
47	Inland	Wenatchee	Chiwaukum	unk.	54	70%
48	Inland	NF Clearwater	Dworshak Hatchery	unk.	68	68%
49	Inland	SF Clearwater	Clear Cr	summer	31	69%
50	Inland	SF Clearwater	Crooked River	summer	86	72%
51	Inland	SF Clearwater	Lolo Cr	summer	69	72%
52	Inland	SF Clearwater	Tenmile Cr	summer	58	66%
53	Inland	Lochsa	Canyon Cr, Deadman Cr	unk.	60	69%
54	Inland	Lochsa	Upper Lochsa R	summer	72	68%
55	Inland	Selway	Hell's Half‐Acre	summer	56	66%
56	Inland	Selway	Little Clearwater R	summer	65	70%
57	Inland	Selway	Upper Selway R	summer	51	68%
58	Inland	Selway	White Cap Creek	summer	72	69%
59	Inland	SF Salmon	Johnson Creek	summer	54	73%
60	Inland	SF Salmon	Lick Creek	summer	70	72%
61	Inland	SF Salmon	Secesh River	summer	30	67%
62	Inland	SF Salmon	South Fork‐East Fork	summer	70	72%
63	Inland	SF Salmon	Stolle Meadows	summer	42	70%
64	Inland	MF Salmon	Bargamin Creek	summer	60	71%
65	Inland	MF Salmon	Big Cr, Rush Cr	unk.	76	72%
66	Inland	MF Salmon	Camas Creek	summer	70	70%
67	Inland	MF Salmon	Loon	unk.	51	71%
68	Inland	MF Salmon	Marsh Cr	summer	51	68%
69	Inland	NF Salmon	East Fork Salmon River	summer	51	73%
70	Inland	NF Salmon	Lemhi R, Bear Valley Cr	unk.	89	70%
71	Inland	NF Salmon	Morgan Cr	summer	74	67%
72	Inland	NF Salmon	Pahsimeroi Hatchery	summer	56	66%
73	Inland	NF Salmon	Sawtooth Hat	summer	47	68%
74	Inland	NF Salmon	West Fork Yankee Fork	summer	58	68%

^a^ Percent of genome covered at a depth of ≥10 reads.

Libraries were created for low‐coverage whole‐genome sequencing from genomic DNA extracted using Chelex (Bio‐Rad). Library preparation followed the individual barcoding protocol from Horn et al. (2020). Briefly, DNA from multiple individuals was normalized and used in a modified version of the Illumina Nextera protocol using indexed primers for each individual fish. Indexed DNA from each individual was pooled equimolarly and sequenced paired‐end, 2 × 150 bp, on an Illumina NextSeq 550 until sufficient pooled genomic coverage was achieved, usually two libraries per instrument run. Based on specifications for this instrument, there was an expected average of 60 Gb of sequence per library with aims of covering at least 50% of the genome with adequate coverage to estimate allele frequencies for each population.

### 2.2 Bioinformatic steps

Reads for each library were trimmed and mapped to the rainbow trout genome (NCBI GCF_002163495.1) using bbmap v04‐11‐2019 and bwa v0.7.17‐r1188 via the *PPalign* module of poolparty2 (Bushnell, 2015; Li & Durbin, 2009; Micheletti & Narum, 2018; Willis et al., 2023). Reads were discarded if trimmed below 50 bp or PHRED quality of 20. Coverage statistics for each library were calculated using the *PPstats* module, using a maximum coverage filter of 1000 reads per collection. A list of segregating variants was generated in the *PPalign* module using bcftools v1.9 (Li, 2011), retaining variants with a PHRED quality of ≥20, more than three bases from an insertion–deletion event, scored in at least one individual in each collection, and with a global minor allele frequency minimum of 0.005. This minor allele frequency filter was applied to exclude artifactual variants (e.g., sequencing errors) without eliminating positions with alleles which segregate in only a subset of stocks, and reflected one allele out of 200 overall, or the equivalent of at least one of 74 collections with more than one‐third of alleles being the minor allele (assuming equal sample sizes). The variant allele counts were tabulated through *PPalign* using popoolations v2_1201 (Kofler et al., 2011), and individual sequence depth was normalized to avoid biased allele frequencies from disproportionate coverage in one or a few individuals in a collection (Figure S1). Allele frequencies were estimated from these normalized counts in R. The final variant set was produced by filtering to eliminate those variants scored in fewer than three individuals or fewer than ten reads per collection and those with more than two segregating nucleotide variants (retaining canonical SNPs), using a combination of the *PPanalyze* module and custom *awk* code.

### 2.3 Statistical analyses

Principal component analysis (PCA) was applied (stats package in R) to observe the population genetic structure represented by all SNPs, as well as two additional PCAs from SNP subsets which exhibited a maximum difference in allele frequencies (MDAF) among collections either above or below 0.9, which we chose to discriminate outlier loci (e.g., trait‐associated or putatively under selection, >0.9) from those that represent the background, putatively neutral genetic structure (<0.9). Neither dataset produced by this segregation was expected to be exclusively ‘adaptive’ or ‘neutral’, respectively, but rather to discriminate sets of loci that are more or less affected by selective versus demographic forces. To compare the effects of this filter and patterns exhibited by SNPs on either side of this cutoff, we also filtered using MDAF cutoffs of higher stringency (MDAF 0.1) and intermediate stringency (MDAF 0.5) and similarly analyzed these data with PCA. To examine how the signal for population structure related to steelhead lineages varied across the genome, we plotted the per‐site *F*_ST_ as well as the mean *F*_ST_ in 100 Kbp windows averaged in steps of 5 Kbp (excluding the lineage‐intermediate sample, Klickitat, no. 11).

We examined patterns reflected by the allele frequency of SNPs within two regions containing loci of large effect on life history phenotypes to ascertain if there was greater variation in allele frequency among collection in these trait‐associated regions compared to the chromosomal background. These regions were a chr. 28 region associated with adult migration phenology (GREB1L/ROCK1; Hess, Zendt, et al., 2016; Micheletti, Hess, et al., 2018; Waples et al., 2022) and a chr. 25 region associated with age at reproductive maturity or first return migration (*SIX6*; Waters et al., 2021; Willis et al., 2020). Variation within each region of chr. 28 and chr. 25 was evaluated by estimating the standard deviation of minor allele frequencies across collections and then calculating the mean of standard deviation in steps of ten kilobases, plotted in a 4 Mbp window centered on known candidate markers, or steps of one kilobase, plotted in a 400 Kbp window. Further, allele frequencies from lcWGS in this study were compared to known candidate markers from chr. 28 (13 SNPs) and chr. 25 (10 SNPs) that were originally identified in genome‐wide association analyses (Hess, Zendt, et al., 2016; Micheletti, Hess, et al., 2018; Prince et al., 2017; Sinclair‐Waters et al., 2020). These candidate SNP markers were previously examined in detail using individual‐level genotype and phenotype data for Columbia River steelhead migrating through the Columbia Basin and sampled at Bonneville Dam (Willis et al., 2020), the first barrier encountered for fish migrating back from the ocean to freshwater spawning grounds. After confirming the appropriate genomic (gene) boundaries based on annotation of the reference assembly, allele frequencies across these genomic regions were plotted for each collection in R to observe patterns of linkage and regional association and consistency with the strength of association (*R* ^2^) of these candidate markers reported by Willis et al. (2020). We also analyzed allele frequencies of these gene‐bounded subregions of chr. 28 and chr. 25 using PCA to examine their distinction from neutral genetic structure, and we plotted the allele frequency of three candidate markers inferred from genomic data. This included the allele frequency for two markers from chr. 28 reflecting two prominent linkage groups, and one marker from chr. 25, for each collection of steelhead to identify patterns in the geographic distribution of allele frequency in these trait‐associated regions. For reference, we also included plots of the probability density function (frequency) of phenotypes, migration day or age at maturity, for each genotype of these three markers using data published by Willis et al. (2020) (https://doi.org/10.5061/dryad.mpg4f4qwt).

Finally, we compared Nei's genetic distance among collections in R (adegenet; Jombart, 2008) from allele frequencies based on lcWGS in the current study versus allele frequencies calculated from individual genotypes (Collins et al., 2020). This comparison enabled a quantifiable approach to verify whether allele frequency estimates from lcWGS reflected patterns in previous studies for both neutral and candidate regions of the genome. These genetic distances also allowed us to evaluate evidence for signals contrasting this neutral pattern in the trait‐associated regions that would imply strong deviation from background genetic structure as a result of discordant natural and/or anthropogenic forces. For putatively neutral variation, genetic distances were estimated from allele frequencies of a subset of putatively neutral SNPs (MDAF < 0.9) from lcWGS and were contrasted with distances among 58 of the same stocks estimated with genotypes of a curated set of 226 neutral SNPs (Collins et al., 2020). To obtain a subset of putatively neutral markers from the whole‐genomic data, a random sample of ten thousand SNPs was taken from each chromosome (total 290 k SNPs with MDAF < 0.9) to conserve computation time. For the two trait‐associated regions on chr. 28 and chr. 25, all of the SNPs from lcWGS in the most strongly associated linkage groups were used (302 and 328 SNPs, respectively) to compare with genetic distance based on individual genotypes from the previous study (Collins et al., 2020). Data for the populations genotyped previously were available on Dryad (https://doi.org/10.5061/dryad.jh9w0vt80) and included many of the same individuals sequenced in this study; further details are available in Collins et al. (2020). The curated SNP markers were genotyped with an amplicon‐sequencing method that has demonstrated high accuracy (Genotyping‐in‐Thousands by Sequencing, or GT‐seq; Campbell et al., 2015), and only individuals with a maximum of 1% missing data were retained. These SNPs reflect a putatively neutral set assembled for their performance in genetic stock identification and parentage‐based tagging applications (Hess et al., 2021).

## 3 RESULTS

Reads per collection ranged from ~160 to 701 million reads, which resulted in between ~59% and ~76% of the genome covered at a minimum of ten reads for each collection (Table 1; Table S1). The *PPalign* module of poolparty2 identified 25.16 million variants that were sampled in at least one individual per collection and were above minimum minor allele frequency of 0.005. The number of SNPs above additional minimum depth and individual requirements was inversely related to the number of collections to which these minima were applied, with 8,066,925 SNPs observed in a minimum of ten reads and at least three individuals in each of 74 collections. Greater numbers of SNPs were retained when filtering within subsets of collections, including a total of 8,893,707 SNPs observed within the 62 Inland lineage collections, and 12,612,115 SNPs observed within the 29 mid‐Columbia collections in reporting group 7. Filtering SNPs based on maximum differences in allele frequency (MDAF) resulted in datasets with differing numbers of SNPs for analyses, such as one with all SNPs (*N* = 8,066,925), a putatively neutral set of SNPs (≤0.9 MDAF; *N* = 8,027,474), and a putatively adaptive set of SNPs (>0.9 MDAF; *N* = 39,451). In contrast, filtering with a maximum MDAF of 0.1 resulted in a large dataset above the cutoff (>0.1 MDAF; *N* = 7,753,600) and smaller dataset below this threshold (≤0.1 MDAF; *N* = 313,325), while filtering with intermediate stringency (MDAF) produced two datasets of intermediate size (1,407,972 and 6,658,953 SNPs > or ≤MDAF 0.5, respectively).

### 3.1 Identifying neutral and non‐neutral structure in whole‐genome sequencing

**FIGURE 2:**
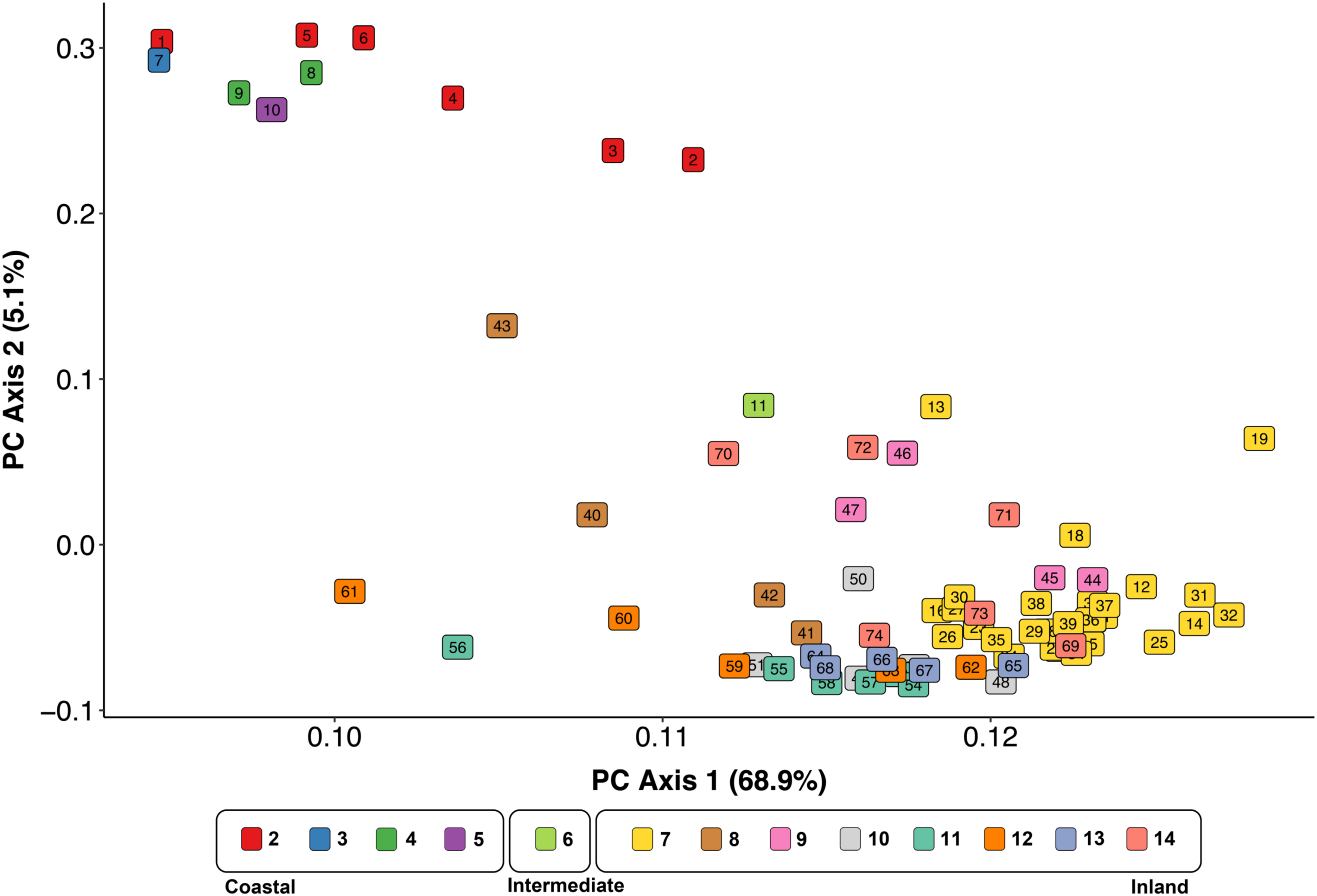
Principal component analysis of allele frequencies from all 8,066,925 SNPs across 74 populations of steelhead. Point labels refer to collections and correspond to Table 1. Colors reflect management reporting groups, which are based on genetic relationships. Reporting groups are labeled and grouped by lineage beneath the plot.

Genetic structure represented via PCA changed depending on the maximum difference in allele frequency (MDAF) allowed among collections (Figure 2; Figure S2). For all 74 collections, SNPs with the largest MDAF were considered outliers (MDAF > 0.9; *N* = 39,451 SNPs), potentially reflecting regions affected by selection as well as drift. Though an MDAF of 0.9, the lowest stringency we applied to exclude outlier loci, is an arbitrary cutoff, this outlier set included a higher proportion of sites from regions of known association with important life history traits on chr. 28 and chr. 25 (Figure S3), indicating that this filter captured the most informative loci in genomic regions under strong influence of selection. The first PC axis in the PCA of these outlier loci was dominated by structure distinguishing the Coastal and Inland lineages and explained ~40% of genetic variance in these SNPs (Figure S2, Panel A). In contrast, the first PC axis for SNPs with MDAF ≤0.9 and reflecting putatively neutral variation (8,027,474 SNPs) showed patterns of clustering between the two major lineages as well as population structure within lineages, which explained ~70% of the genetic variance from these data (Figure S2, Panel B). The population structure reflected by this putatively neutral set was nearly identical to patterns inferred without allele frequency filtering (all 8,066,925 million SNPs; Figure 2). The datasets produced by filtering with intermediate stringency (MDAF 0.5) largely reflected a similar pattern to lower stringency (MDAF 0.9) (Figure S2, Panels C, D). The putatively neutral SNPs from the most stringent filter (MDAF ≤ 0.1; 313,325 SNPs) only reflected structure among collections within Coastal and Inland lineages (Figure S2, Panel F), while the set excluded by this filter, containing both the outlier and majority of putatively neutral SNPs (>0.1 MDAF; 7,753,600 SNPs), exhibited a pattern much like the unfiltered dataset and those between MDAF of 0.9 and 0.1 (Figure S2, Panel E). These results suggest that while an MDAF threshold of 0.9 to exclude outliers is arbitrary and may miss some sites affected by selection, outlier SNPs have relatively little influence on the pattern of population structure exhibited by the majority of SNPs. Plots of *F*_ST_ similarly indicated that the main signal for population structure was distributed across most of the genome rather than being concentrated in a few genomic regions (Figure S4).

### 3.2 Variation at major effect loci relative to neutral variation

**FIGURE 3:**
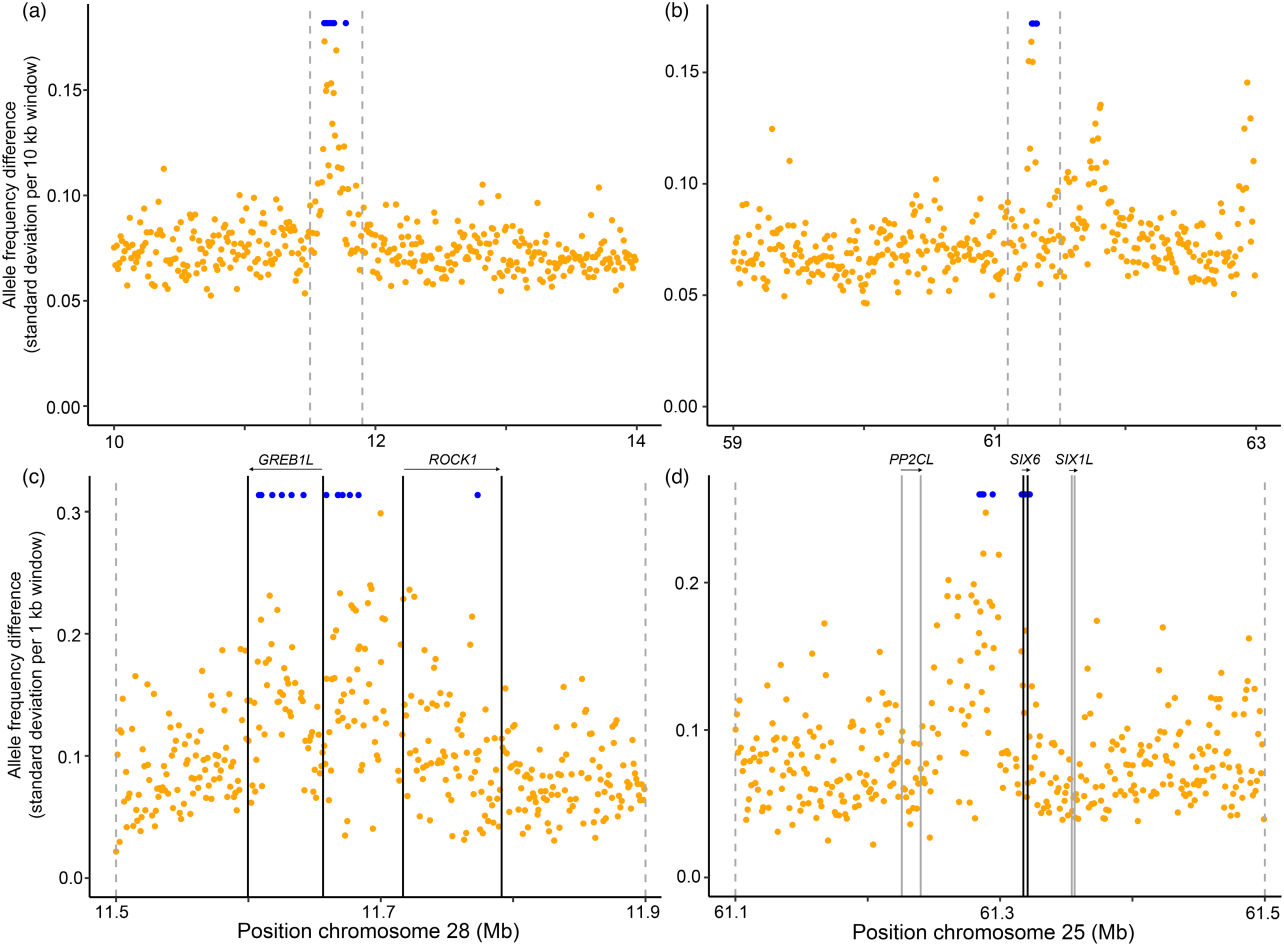
Allele frequency differences across collections of steelhead, estimated as mean of standard deviations within windows, for genomic regions associated with life history trait variation. Panels A and C show chromosome 28 at broad and narrow scale, and panels B and D show chromosome 25 at broad and narrow scale. The dashed line indicates the same plot area in either the left (chr. 28; a,c) or right (chr. 25; b,d) panel pair. Note the difference in scale for 10 Kb windows (a,b) and 1 Kb windows (c,d); In each panel, genomic positions for existing GT‐seq markers are indicated in blue relative to other SNPs within each window (yellow), but y‐axis values are arbitrary. The outer boundaries of the depicted gene regions (*GREB1L‐ROCK1* and *PP2CL‐SIX1L*) were used for plotting allele frequency in Figure 4.

**FIGURE 4:**
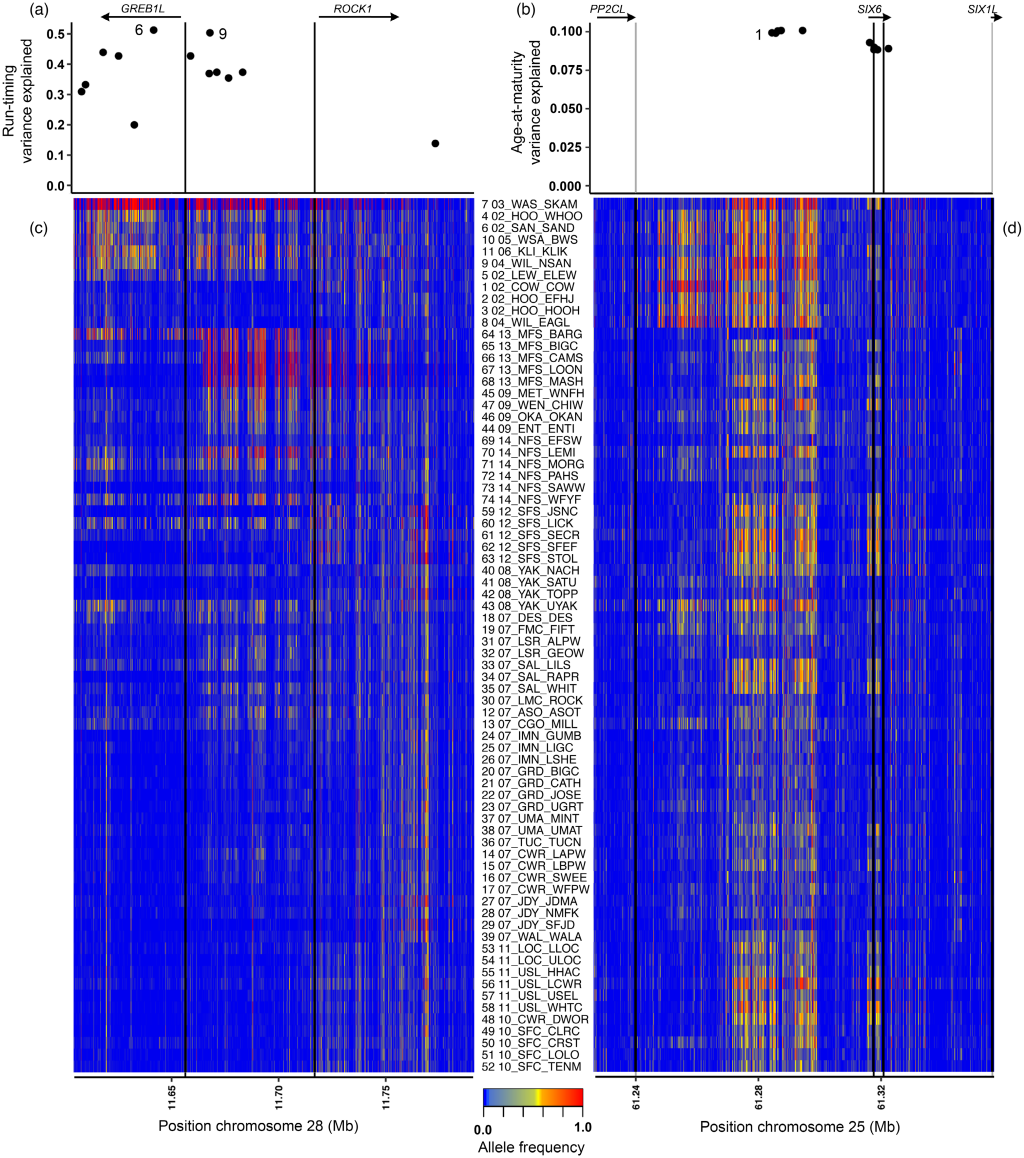
Allele frequencies in each steelhead collection for SNPs in the regions of strong association with life history trait variation. For each of the regions of chr. 28 (migration phenology) or chr. 25 (age at maturity), the phenotypic variance explained by markers developed for GT‐seq is shown in panels a and b (from Willis et al., 2020) along with the relative position of SNPs plotted in Figure 5 are indicated (chromosome 28: 6, 11,641,623 bp; 9, 11,667,915 bp; chromosome 25: 1, 61,284,413 bp) while allele frequency in each steelhead collection for the positions surveyed with whole‐genome sequencing are indicated by color in panels c and d. Samples are ordered within lineage according to run timing reported by Hess, Ackerman, et al., (2016); Hess et al., (2023).

For the trait‐associated regions on chr. 28 (adult migration phenology) and chr. 25 (age at maturity), the mean standard deviation of allele frequency across samples in windows of SNPs near previously identified candidate markers confirmed that the most variable regions among collections were adequately captured by the boundaries of genes residing in or nearby the regions of strongest trait association (Figure 3). Notably, variation in allele frequency between collections was not consistent across lineages, as multiple linkage groups were evident, and the boundaries of these linkage groups corresponded to changes in the strength of association in candidate SNP markers seen in Willis et al. (2020) (Figure 4). Notably, for chr. 28, there was a strong distinction in allele frequency variation among samples between a subregion containing *GREB1L* (as well as a small portion of the intergenic region immediately adjacent to *GREB1L*, containing candidate markers 1–7; hereafter the *GREB1L* subregion) versus the central subregion containing most of the intergenic sites (containing candidate markers 8–12; hereafter the *GREB1‐ROCK1* intergenic subregion), the former of which contains the most strongly associated positions (Willis et al., 2020). Similarly, there was a distinction in the degree of variation across the chr. 25 region examined, with much greater variation in the central *PPC2L‐SIX6* intergenic region plus the *SIX6* gene itself, which contain the most strongly associated positions (Willis et al., 2020).

Principal component analysis of SNPs within these chr. 28 and chr. 25 regions of large effect reflected the complexity in allele frequency and linkage across populations and lineages (Figure S5). Notably, the collection from the Skamania Hatchery stock, a stock in the Coastal lineage that has a history of strong artificial selection for older (2+ ocean years) and early (spring–summer) migrating fish, typified the extreme of one PC axis in each analysis (Figure S5, collection number 7). Populations with higher frequencies of the allele associated with early adult migration (chr. 28, Figure S5, Panel A) or longer ocean duration (chr. 25, Figure S5, Panel B) clustered more closely to the Skamania Hatchery collection, and those with lower frequencies were distinct.

**FIGURE 5:**
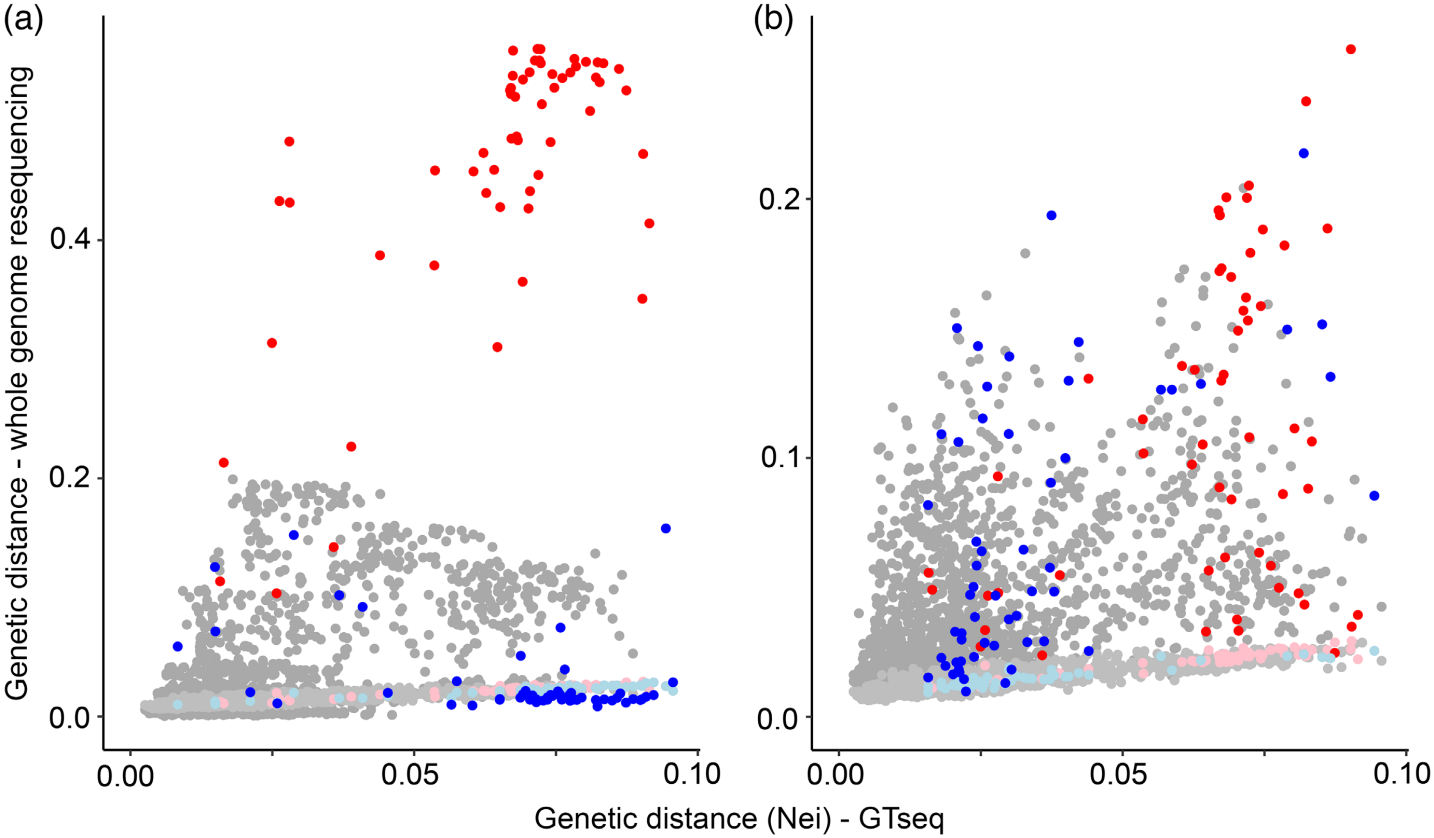
Nei's genetic distance among pairs of 54 steelhead collections from genomic resequencing data versus GT‐seq (amplicon sequencing). On the x‐axis of both panels are plotted genetic distances from allele frequencies calculated from genotypes of 226 curated, putatively neutral SNPs surveyed with GT‐seq (original data from Collins et al., 2020). On the y‐axis of both panels are plotted genetic distances for the same stocks calculated from allele frequencies from a sample of putatively neutral SNPs (MDAF < 0.9) on each chromosome in the whole‐genomic data (lighter shaded dots); most localities are in gray, while two select samples in each panel are highlighted in light blue (Skamania in both panels) or pink (Cowlitz River in panel a and Bargamin Creek in panel b). Also plotted in darker shades on the y‐axis are distances of the most strongly trait‐associated linkage groups of chr. 28 (panel a) or chr. 25 (panel b); again, most localities are plotted in gray, while distances for the same highlighted samples are plotted in blue and red, respectively.

There was a strong linear association of Nei's genetic distance among populations with the two sets of SNP markers (Spearman's *r* ^2^ = 0.855) when comparing genetic distances calculated from allele frequencies of putatively neutral genomic SNPs (a random sample of 290 k of the 8,027,474 million SNPs from MAFD ≤ 0.9) against distance calculated from allele frequencies estimated from genotypes of 226 curated SNP markers for 54 of the same populations of steelhead (Collins et al., 2020). Notably, this correlation was similar to the correlation with the unfiltered genomic dataset (0.860) but higher than datasets filtered with a MAFD ≤ 0.5 (0.714) or ≤0.1 (0.164). This pattern supported the expectations of neutral variation between the two data sets, including for the Skamania Hatchery stock, although the value of genetic distance was an order of magnitude larger with the curated panel of 226 SNPs than the genomic data (Figure 5). From this, we inferred that the allele frequencies estimated from lcWGS represented the same background neutral genetic population structure as observed in previous studies using other genetic technologies (Blankenship et al., 2011; Collins et al., 2020; Hess et al., 2021; Micheletti, Matala, et al., 2018). In contrast, when genetic distance for the linkage groups containing SNPs of strongest association in each large effect locus (Figure 4) of chr. 28 (11,599,896–11,667,000 bp) and chr. 25 (61,275,800–61,320,837 bp) was compared against that from the 226 curated, genotyped markers, the linear association was no longer evident (darker shaded points in Figure 5). Instead, SNPs within the trait‐associated regions exhibited notably lower or higher divergence than the neutral genetic distances depending on the trait and collections involved, indicating that the patterns exhibited by these two trait‐associated regions are influenced by distinct additional processes than those that produced the background genetic structure.

### 3.3 Distinct processes influencing variation at major effect loci

**FIGURE 6:**
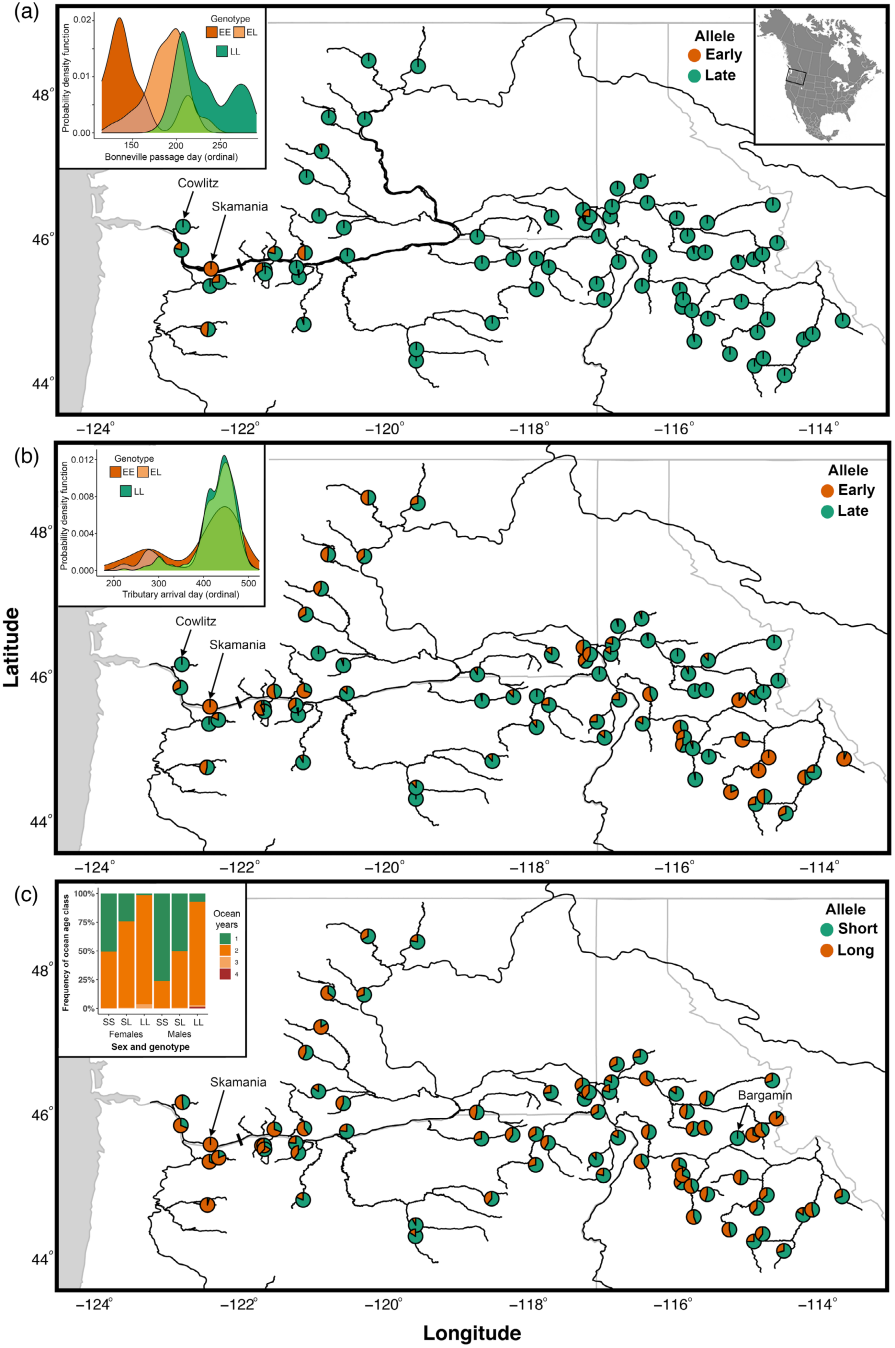
Allele frequencies in steelhead collections of select markers associated with life history trait variation (chr. 28, early vs. late migration timing; chr. 25, long vs. short ocean duration). Pie chart colors of allele frequencies correspond to the phenotype with which they are associated in the inset diagrams. Marker designations and positions are indicated in Figure 4. Insets are modified from Willis et al. (2020) using phenotypic and genotypic data collected from steelhead migrating across Bonneville Dam, the first barrier for inland migrants (indicated by solid bar). (a) frequencies of alleles associated with early (E) or late (L) migration day across Bonneville Dam (a proxy for freshwater entry) on chromosome 28 (marker 6, SNP located at 11,641,623 bp) from the *GREB1L* subregion; inset: probability density function (proportion across time) of migration day for Inland lineage individuals with each genotype. (b) frequencies of alleles associated with early (E) or late (L) date of arrival to spawning tributary on chromosome 28 (marker 9, SNP located at 11,667,915 bp) from the *GREB1L‐ROCK1* intergenic subregion; inset: probability density function of arrival date for Inland lineage individuals by genotype. (c) frequencies of alleles associated with short (S) or long (L) ocean duration on chromosome 25 (marker 1, SNP located at 61,284,413 bp) from the *PP2CL‐SIX6* intergenic subregion; inset: proportion of individuals of a given ocean age (duration) for Inland lineage individuals, separated by sex, for each genotype.

Genetic distances in trait‐associated regions indicated several selective processes that have acted on these regions, and comparisons of the Skamania Hatchery stock with two other Columbia basin stocks illustrate this point (Figure 5). Specifically, the Skamania Hatchery stock, which exhibits distinctly early migration timing, was strongly divergent in the chr. 28 trait‐associated region from all other collections in the study that had a high frequency of alleles associated with later migration timing, including Coastal lineage stocks to which it is otherwise genetically similar at neutral SNPs. In contrast, a typically late migrating stock in the Coastal lineage, the winter‐run collection of the Cowlitz River, a tributary of the Columbia River west of the Cascades, was more similar to Inland lineage collections at chr. 28 trait‐associated markers than with the neutral SNPs, since the Inland lineage stocks have high frequencies of alleles associated with late migration but are otherwise highly divergent from Coastal lineage stocks. At the same time, the Cowlitz collection was quite divergent in chr. 28 markers from early migrating Coastal lineage stocks to which it was otherwise more genetically similar at neutral SNPs. This pattern was also evident in maps of allele frequency for early and late migration‐associated alleles at the strongest candidate markers (Figure 6): the frequency of early migration alleles in the *GREB1L* region was rare across Columbia Basin collections except for the Skamania Hatchery collection that has experienced artificial selection for early migration and those nearby that have had stocking or strays from this lineage. Notably, however, variation was higher between collections across the basin for the *GREB1L‐ROCK1* intergenic linkage block of chr. 28 (Figure 6), a region that may be associated more strongly in Inland stocks with arrival timing at spawning grounds or other aspects of migration besides freshwater entry (Micheletti & Narum, 2018; Willis et al., 2020; Figure 6). Similarly, the region on chr. 28 where there were the largest differences in allele frequencies was substantially smaller in the Inland lineage (~82 Kb) relative to the Coastal lineage (~117 Kb; Figure 4). In particular, populations in the Coastal lineage had strong differentiation that extended into the *GREB1L* coding sequence but those from the Inland lineage did not.

The Skamania Hatchery stock, which consists of fish with distinctly older age at maturity, was also noticeably divergent in the chr. 25 region from stocks with higher frequencies of alleles associated with younger age at maturity, despite geographic proximity and similarity in neutral SNPs (Figure 5). Conversely, the Bargamin Creek collection, from an Inland lineage population in the middle Salmon River of Idaho with a high frequency of alleles associated with younger age at return, was more similar in chr. 25 variation to otherwise more genetically distant (and less geographically proximate) collections than with nearby populations. In contrast to the strongest trait‐associated sites of chr. 28, a geographic plot of the allele frequency of the strongest candidate marker in this chr. 25 region showed that variations for alleles associated with age at maturity were highly variable within and among populations of both lineages (Figure 6). For both trait‐associated regions in chr. 28 and chr. 25, the extent of SNPs with the largest differences in allele frequencies was also narrower across populations in the Inland lineage (~27 Kb) relative to the Coastal lineage (~52 Kb; Figure 4). Specifically, populations in the Coastal lineage had strong differentiation that extended further upstream from the *SIX6* gene into the *PP2CL‐SIX6* intergenic region than those from the Inland lineage (Figure 4). Nonetheless, there was more intermediate and less discrete variation among collections along chr. 25 for populations of the Inland lineage than was evident for chr. 28, indicating a distinct history and scale of selective processes governing local allele frequencies.

## 4 DISCUSSION

Several previous studies have examined genetic variation in salmonid populations using low‐to‐medium marker density, but here we utilized a dataset of whole‐genome sequencing data from dozens of collections to examine genomic patterns of differentiation in steelhead trout populations throughout the Columbia River Basin. Our use of a dedicated pipeline and targeted filtering produced a large variant library that will provide a highly useful resource for those studying steelhead, including over 8 million SNPs for which allele frequency information could be inferred for all of our 74 collections, and for various subsets of these collections (e.g., >12 million SNPs from collections representing reporting group 7). While it is important to clarify that the allele frequencies from low‐coverage sequencing may be less accurate than higher coverage sequencing for the same number of individuals (Fuentes‐Pardo & Ruzzante, 2017; Lou et al., 2021), our study verified that estimates from lcWGS were able to capture similar patterns of genomic differentiation as previous studies with neutral markers and trait‐associated loci (e.g., Collins et al., 2020; Willis et al., 2020).

### 4.1 Neutral variation from whole‐genome sequencing

In this study of Columbia River steelhead, we observed that genetic variation reflected by putatively neutral sites (99.5% of SNPs) was consistent with patterns of structure discovered from the targeted markers genotyped by previous studies (Blankenship et al., 2011; Collins et al., 2020; Hess et al., 2021; Micheletti, Matala, et al., 2018). Rainbow trout and their anadromous conspecifics, steelhead, are known to exhibit extensive phylogeographic and population structure, reflecting the influence of both geological/glacial and diverse landscape‐level selective forces in shaping contemporary genetic structure (Blankenship et al., 2011; Micheletti, Matala, et al., 2018; Waples et al., 2008). Identification of this genetic structure has been important not only for understanding the evolutionary history and designation of conservation units (e.g., Waples, 1995) but also for distinguishing the contributions of distinct stocks to mixed fisheries and tracking the fate of imperiled populations of various species (Funk et al., 2019; Moore et al., 2021; Utter & Ryman, 1993), including steelhead (e.g., Hess, Ackerman, et al., 2016).

Notably, the majority of previous studies have utilized discrete sets of curated molecular markers that have transitioned over decades from allozymes, mitochondrial DNA, microsatellites, and more recently select SNP markers (e.g., Allendorf, 2017). The advantage to focused sets of markers is their ability to be utilized in studies across labs and therefore comparability among separate studies of these species, but ultimately these reflect the variation of only a miniscule subset of the genome. In contrast, the data utilized here reflect over 8 million variants distributed across the majority of the genome and recovered the same lineage structure and a strong linear correlation between genetic distances among populations in this study compared to previous results (Collins et al., 2020), but genetic distances estimated using this neutral genomic variation were notably an order of magnitude lower than those from a set of targeted SNPs surveyed with GT‐seq. This not only reflects a pattern that often occurs among different marker sets in many species (e.g., Zimmerman et al., 2020) but also more accurately conveys the true degree of neutral genetic divergence among stocks. Notably, SNPs that were putatively under selection (39,451 SNPs) did not skew the overall population structure inferred from the combined set of all 8,066,925 million SNPs, which largely reflected the same patterns as the putatively neutral data (8,027,474 SNPs). Although there were many more neutral than outlier SNPs, this result was also because the variance explained by the neutral SNPs was more concentrated in the first PCA axis, while variance explained by the fewer outlier SNPs was more evenly distributed among several axes when these were analyzed separately. This suggests that neutral SNPs reflected a more cohesive pattern of genetic structure influenced similarly by historical and demographic forces, in contrast to the myriad of overlapping and potentially conflicting selective forces that have influenced the allele frequencies of outlier regions.

### 4.2 Variation at major effect loci is distinct relative to neutral variation

We observed that two regions of known association with life history variation in steelhead indeed reflected distinct patterns of allele frequency variation relative to the putatively neutral SNPs we surveyed with these data. For instance, while early and late migrating steelhead often spawn in proximity in several subbasins, most collections appear to be strongly skewed in allele frequency toward the most common migration phenology in each population. This suggests that adult migration timing is under directional selection within most tributaries, perhaps because distinct environments favor certain migration phenology extremes (early or late migration and/or spawning) or because of positive‐density dependence in reproductive success, most conceivably in stocks with limited abundance (Quinn et al., 2016). As a result, while genetic distance of the neutral loci approximately followed geographic proximity of populations, in many instances collections from across the basin were more similar to each other at trait‐associated loci than were collections from nearby populations. Notably, not only was the variation in allele frequency among collections considerably larger in magnitude in the trait‐associated regions than the variation in neutral loci, but the patterns represented by each region were highly distinct as well. Indeed, the patterns of variation at each of these trait‐associated regions differed as much from each other as from the neutral genetic variation, implying different historical and/or contemporary forces shaping the distribution of genetic variation. These patterns indicate that local natural selection is distinct from the effects of gene flow and demography alone, and selects for distinct combinations of phenotypes associated with each of these two large effect regions.

Importantly, while most Inland lineage samples were fixed for the allele associated with late migration timing, fish from these interior populations migrate earlier (July through November) than their coastal counterparts with the same alleles, which arrive during the winter months prior to spawning in the spring. Nonetheless, there is still notable variation within the earlier migration period of Inland lineage fish which has been broadly associated with this chr. 28 *GREB1L‐ROCK1* region (Micheletti, Hess, et al., 2018; Willis et al., 2020), potentially indicating that there are as yet unidentified genomic variants that mediate the effect of this genomic region on run timing in this lineage (Willis et al., 2020). Moreover, it is unknown if variation in the different linkage groups in this region has the same effect on different aspects of migration timing such as the date of freshwater entry versus the date of arrival to spawning grounds (Micheletti, Hess, et al., 2018; Willis et al., 2020). However, arrival timing to spawning grounds is more likely to be subject to stock‐specific challenges like physical and thermal barriers to migration (Keefer et al., 2008; Keefer, Clabough, et al., 2018), potentially making genetic correlation harder to assess. This is consistent with the patterns of linkage and population variation across the *GREB1L‐ROCK1* region, which shows much greater variation among Inland lineage fish in the intergenic subregion than the *GREB1L* subregion (Micheletti, Hess, et al., 2018; Willis et al., 2020). While these results add intriguing details to the puzzle of genomic architecture of migration phenology, determining the full extent of association of variants in this chr. 28 region and others will depend on sufficiently dissecting phenotypic variation within multiple aspects of anadromous migration, including migration staging, arrival timing, and smolt out‐migration.

In contrast to patterns of variation observed for migration phenology, the allele frequencies in the region on chr. 25 most associated with age and maturity were more intermediate and varied more continuously across collections, consistent with the mixtures in ages seen in most stocks (Hess, Ackerman, et al., 2016). However, there were notable exceptions such as populations with high frequency of ‘long‐duration’ alleles in the Clearwater River and Salmon River subbasins, located in northwestern and central Idaho, respectively, that are known for exhibiting predominantly 2 + −ocean migrants (Willis et al., 2020). In contrast, Bargamin Creek, a tributary of the Salmon River, exhibited allele frequencies strongly skewed toward alleles associated with shorter ocean duration. This suggests selection may favor distinct life history suites that include age at maturity at relatively small geographic scales in certain regions, while balancing selection maintains variation in age classes within most populations (e.g., Barson et al., 2015).

### 4.3 Anthropogenic processes affecting variation at major effect loci

In addition to the natural forces that have produced geographic and structural signatures on genetic variation in the trait‐associated regions of chr. 28 and chr. 25, anthropogenic processes have left clear impacts as well, in the form of more extreme phenotype distributions and allele frequencies than produced by natural selection alone. In the example highlighted in this study, the allele frequency and linkage patterns in the large effect regions of chr. 28 and chr. 25 are clearly distinct from the natural origin stocks, reflecting the opportunity for management actions to have long‐term effects on the genetic and phenotypic diversity of exploited species.

One of the most important and controversial management practices in the Columbia River Basin is the use of fish hatcheries to supplement diminishing natural stocks of anadromous salmonids including steelhead (Paquet et al., 2011). Among the many hatchery programs in the basin, Skamania Hatchery is notable for the phenotypic distinctness of the steelhead produced by this hatchery, a result of both intentional and inadvertent selective breeding and culling over decades. Indeed, the earliest management period for steelhead returning to the Columbia Basin (April 1 to June 30) is known as the ‘Skamania management period’ because of the high proportion of early migrants from this hatchery (ODFW/WDFW, 2022). Ayerst (1976) describes the intentional selection of steelhead broodstock for fish of larger size (known at the time to be most often two‐ or three‐ocean fish) and fish that were ready for egg harvest at an earlier time. Egg harvest at Skamania Hatchery was initially in late March and had been advanced to February 1976 but has been shifted to as early as December in recent decades (Hess et al., 2020). Thus, while selection for longer ocean‐duration fish was intentional toward the goal of producing larger fish, the distinctly early return time for Skamania Hatchery migrants was apparently an unintentional by‐product of selecting fish that are ready to spawn earlier the following year. This selection has left clear signatures on the genomic regions now known to be associated with these traits, on chr. 28 and chr. 25, producing both a decrease in genetic diversity around the markers with the greatest trait associations and an increase in linkage disequilibrium across the whole trait region due to genetic hitchhiking. Though perhaps not to the same degree, this same kind of selective breeding has been applied at other Columbia River basin hatcheries, and until relatively recently, avoiding hatchery returns in favor of natural origin fish as broodstock was not routine practice at most hatcheries (e.g., Horn et al., 2023; Paquet et al., 2011), creating additional opportunity inadvertent anthropogenic selection (Ayerst, 1976; Busby et al., 2000; Crawford, 1979).

In addition to selection on hatchery stocks themselves, Skamania Hatchery steelhead have been outplanted in many places throughout the Columbia River Basin to mitigate dwindling populations, sometimes without fully accounting for the suite of life history traits or genetic integrity of the native stock. The result has been introgression of distinct variation and modified allele frequency in chr. 28 and chr. 25 through introduction of the Skamania Hatchery stock into other Columbia River Basin stocks such as those in the lower and middle regions of the Columbia River. In other cases, Skamania Hatchery fish have been known to stray into other tributaries in the lower Columbia River leading to introgression (Hess et al., 2020).

While our collections focused on anadromous stocks, another source of anthropogenic selection to which our data could be applied is the genetic influence of rainbow trout (resident, non‐anadromous forms of *O. mykiss*), which will interbreed with anadromous migrants when possible (Kendall et al., 2015 and references therein). Residency in steelhead has been extensively studied and is believed to be influenced by genetics, environment, condition, and sex, but the trade‐off for traits expressed in resident versus anadromous forms is an active area of research (Collins et al., 2022; Kendall et al., 2015; Leitwein et al., 2017; Pearse et al., 2019). Like some anadromous hatchery stocks, extensive outplanting of non‐local stocks of rainbow trout across the Columbia River Basin is likely to have influenced the native genomic variation for life history traits examined here, with unknown impacts on proximal anadromous stocks. Conversely, undisturbed populations of resident rainbow trout may provide reservoirs of native genetic variation (Andrews et al., 2023) for recovery of anadromous stocks highly impacted by stocking‐ or straying‐facilitated introgression of non‐local hatchery stocks, if the necessary connectivity between resident and anadromous stocks was available through natural or mediated actions (Chen et al., 2022). Without collections of nearby resident populations, we can only speculate on these effects, but the current dataset presents an opportunity for examining variation at these and other genomic regions when those collections become available. Nonetheless, it is clear that even anadromous steelhead in the Columbia Basin migrate back to natal reaches with heterogeneous environmental characteristics, including annual flow regime, temperature, chemistry, turbidity, elevation, and rainfall, in addition to distinct challenges in their migration paths. Examining loci that may contribute to overcoming these challenges among distinct stocks will require considerable effort, but the present dataset provides a unique opportunity to begin identifying the genomic regions associated with this spatiotemporal variation (e.g., Micheletti, Matala, et al., 2018).

### 4.4 Inferring population genomic structure from low‐coverage data

Massively parallel or ‘next‐generation’ sequencing has dramatically decreased the cost of surveying genetic variation across statistically meaningful numbers of individuals. However, sequencing the complete genome of each surveyed individual at high coverage is not yet practical, in part because as the cost of sequencing has decreased, demands for statistically robust sample sizes have become more ardent (e.g., Schlötterer et al., 2014). As a result, geneticists are still faced with the task of determining the appropriate compromise between number of reads devoted to the extent of the genomic survey of each individual and the number of individuals surveyed (Lou et al., 2021). Many researchers have addressed this compromise by surveying only a directed subset of the genome (e.g., Baird et al., 2008), but, while these techniques provide data on many more loci than what was historically accessible, only a small fraction of the genome is typically sampled, and many linkage blocks may not be included (Hoban et al., 2016). As a result, investigations that only survey a minor subset of linkage groups may fail to identify loci associated with features of interest in many cases (Lowry et al., 2017; Tiffin & Ross‐Ibarra, 2014). On the other hand, because of sampling variance, sampling more individuals (chromosomes) at low coverage actually provides more accurate estimates of population or phenotype allele frequencies than sequencing fewer individuals at high coverage, provided that all included individuals are representative of the intended phenotype or population (Futschik & Schlötterer, 2010; Günther & Coop, 2013; Schlötterer et al., 2014; Zhu et al., 2012). Thus, many population genetic analyses may be performed more effectively by low‐coverage whole‐genome sequencing than with methods that provide individual genotypes, since allele frequencies can be estimated with superior accuracy to higher coverage surveys of fewer individuals (Lamichhaney et al., 2012; Lou et al., 2021; Schlötterer et al., 2014; Therkildsen & Palumbi, 2017).

Our approach with individually barcoded samples and use of a bioinformatics routine that normalized the contribution to allele frequencies of varying read coverage across individuals largely eliminated the variance introduced by varying sample representation among loci (Micheletti & Narum, 2018; Willis et al., 2023). Indeed, a recent examination of the bioinformatic pipeline applied here, poolparty2, using simulated data reflecting the coverage and variation in individual representation of the empirical data utilized in the present study showed that this pipeline showed remarkable accuracy in identifying true segregating sites (≥99.5%) and estimating their allele frequency (≥0.84 Spearman's rank correlation) (Willis et al., 2023). Moreover, our analytical focus on patterns represented by contiguous regions of the genome rather than individual variants minimizes the effects of idiosyncrasies of read depth, since there is no reason to expect them to affect many loci systematically (Schlötterer et al., 2014). From these data, we made an unprecedented examination of the degree and pattern of variation across the genome, including at genomic regions associated with life history traits that have been shaped by discordant selective forces, and the full suite of genome‐wide variation will be critical to ensuring the persistence and ecological services of these populations (e.g., Kardos et al., 2021).

## 5 CONCLUSIONS

This study provides insight into the influences of genomic variation on an ecologically, culturally, and economically important species. By utilizing whole‐genome sequencing data of steelhead populations across a highly heterogeneous landscape, we were able to estimate the pattern and magnitude of putatively neutral variation, characterize variation in genomic regions known to be associated with life history traits critical to local adaptation, and identify how effects of anthropogenic forces differed from the influences of natural selection and historical population demography. We expect these data will represent an ongoing, important resource for understanding many factors affecting persistence and ecological services in these iconic migratory fishes.

## CONFLICT OF INTEREST STATEMENT

The authors assert no conflicts of interest in the publication of this article.

## Supporting information


Figure S1.

Figure S2.

Figure S3.

Figure S4.

Figure S5.

Table S1.

## Data Availability

Data referenced in this manuscript are available from NBCI Short Read Archive (PRJNA854899). The pipeline used for bioinformatic processing of these data, poolparty2, is available from GitHub (https://github.com/stuartwillis/poolparty).
