# Supplementary figures and images for: Contrasting patterns of sequence variation in steelhead populations reflect distinct evolutionary processes
Source: Evol Appl. 2023 Dec 11;17(1):e13623. doi: 10.1111/eva.13623 (PMC10810252; doi:10.1111/eva.13623)

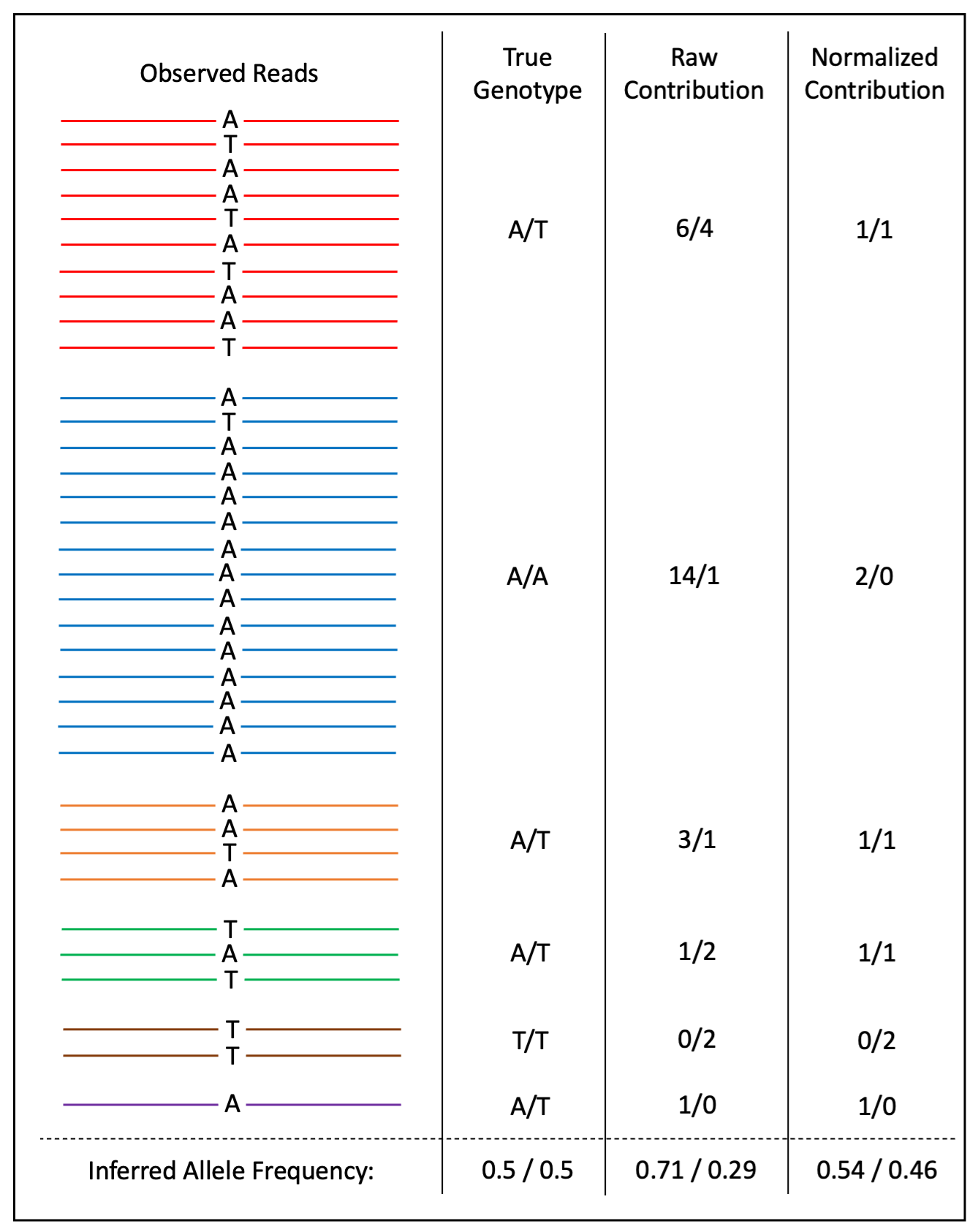

Supplement: Supplementary file 1 — Figure S1. [file EVA-17-e13623-s005.tiff]

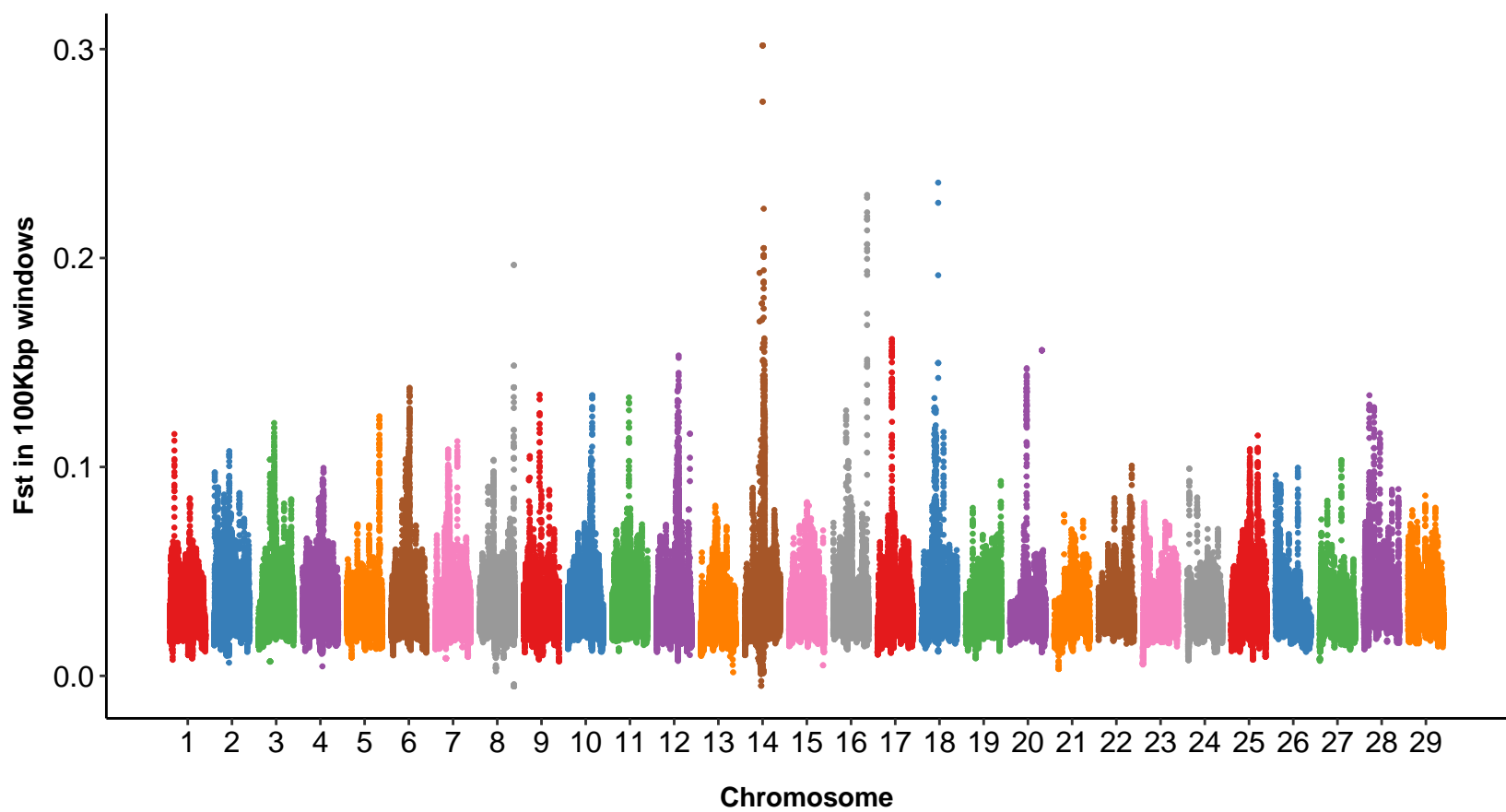

Supplement: Supplementary file 4 — Figure S4. [file EVA-17-e13623-s002.pdf]

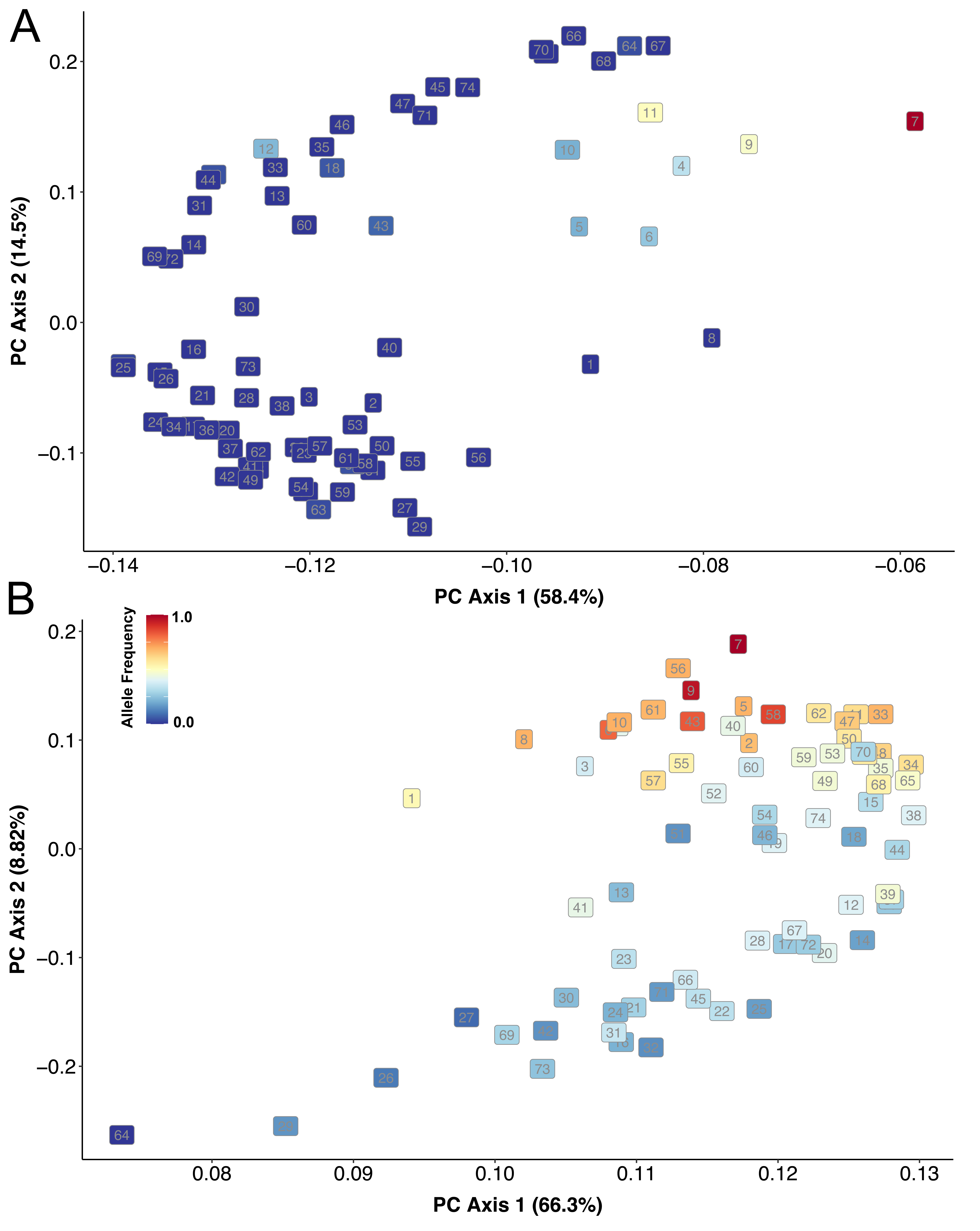

Supplement: Supplementary file 5 — Figure S5. [file EVA-17-e13623-s004.tif]
